# Hypothalamic KLF4 mediates leptin's effects on food intake via AgRP

**DOI:** 10.1016/j.molmet.2014.04.001

**Published:** 2014-04-15

**Authors:** Monica Imbernon, Estrella Sanchez-Rebordelo, Rosalia Gallego, Marina Gandara, Pamela Lear, Miguel Lopez, Carlos Dieguez, Ruben Nogueiras

**Affiliations:** 1 Department of Physiology, CIMUS, University of Santiago de Compostela-Instituto de Investigación Sanitaria, Santiago de Compostela 15782, Spain; 2 CIBER Fisiopatología de la Obesidad y Nutrición (CIBERobn), 15706, Spain; 3 Department of Morphological Sciences, School of Medicine, University of Santiago de Compostela-Instituto de Investigación Sanitaria, Santiago de Compostela 15782, Spain

**Keywords:** AgRP, Food intake, Leptin

## Abstract

Krüppel-like factor 4 (KLF4) is a zinc-finger-type transcription factor expressed in a range of tissues that plays multiple functions. We report that hypothalamic KLF4 represents a new transcription factor specifically modulating agouti-related protein (AgRP) expression *in vivo*. Hypothalamic KLF4 colocalizes with AgRP neurons and is modulated by nutritional status and leptin. Over-expression of KLF4 in the hypothalamic arcuate nucleus (ARC) induces food intake and increases body weight through the specific stimulation of AgRP, as well as blunting leptin sensitivity in lean rats independent of forkhead box protein 01 (FoxO1). Down-regulation of KLF4 in the ARC inhibits fasting-induced food intake in both lean and diet-induced obese (DIO) rats. Silencing KLF4, however, does not, on its own, enhance peripheral leptin sensitivity in DIO rats.

## Graphical abstract

**Figure:**
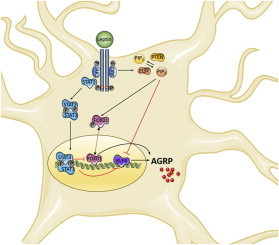

## 1 Introduction

Krüppel-like factor 4 (KLF4) is a zinc-finger-containing transcription factor that binds to GC-rich DNA with a consensus binding sequence of CACCC [1,2]. KLF4 contains both transcriptional activation and repression domains, and thereby activates and represses gene expression [3]. KLF4 regulates multiple biological functions, including cell proliferation and differentiation. Its importance is highlighted by the fact that its germ-line deletion leads to postnatal lethality due to severe dehydration [4]. KLF4 is expressed predominantly in the gastrointestinal tract (especially the colon and small intestine), but also in a wide range of cell types [5]. In the brain, KLF4 is expressed in neural stem cells [6] and is critical to neuronal differentiation [7]. Recent evidence suggests that KLF4 also plays an important role in the central regulation of energy balance. *In vitro* studies show that KLF4 is a transcriptional regulator of agouti-related protein (AgRP) and that pharmacological activation of AgRP is abrogated when KLF4 is down-regulated [8]. Accordingly, GT1-7 cells transfected with a mouse KLF4 construct show a significant increase in AgRP message expression [9].

AgRP neurons are located within the hypothalamic arcuate nucleus (ARC), which contains two neural populations that are crucial to the regulation of food intake. In one of these populations, neurons co-express neuropeptide Y (NPY) and AgRP, both potent stimulators of food intake, while an adjacent set of ARC neurons co-express proopiomelanocortin (POMC) and cocaine- and amphetamine-regulated transcript (CART), both of which suppress food intake [10]. The cells of both populations respond to signals pertaining to long- and short-term energy status in the animal. The fact that AgRP neuronal projections are mostly restricted to brain areas involved in energy balance has made AgRP an attractive target for pharmacological inhibition in relation to energy balance. Numerous reports postulate that AgRP neurons are essential to the hyperphagic response: central administration of AgRP stimulates feeding [11], ablation of AgRP neurons suppresses food intake [12–14], and optogenetic [15] or pharmacogenetic [16] stimulation of cells expressing AgRP induces feeding. Genetic deletion of AgRP neurons, however, has only a slight effect on energy balance, which has been attributed to developmental compensation [17]. Indeed, post-embryonic ablation of AgRP neurons leads to a marked decrease in food intake, probably due to the resulting dysregulation in GABA signalling [18,19].

The *in vivo* regulation of AgRP neurons depends on several transcription factors including forkhead box protein 01 (FoxO1) [20], uracil nucleotide/cysteinyl leukotriene receptor (Gpr17) [21], the hypothalamic homeobox domain transcription factor Bsx [22], signal transducer and activator of transcription 3 STAT3 [23] and diencephalon/mesencephalon homeobox 1 (Dmbx1) [24]. Since *in vitro* studies show that KLF4 can directly control AgRP promoter activity [8], we sought to investigate the *in vivo* role of this transcription factor. Using gain- and loss-of function virogenetic approaches, we have demonstrated that induction of KLF4 in the ARC increases feeding behaviour and weight gain through the specific stimulation of AgRP. Over-expression of KLF4 in the ARC was sufficient to completely block the suppression of caloric intake and weight gain caused by leptin in lean rats. Consistent with those data, genetic inhibition of KLF4 in the ARC of lean and high fat diet (HFD)-fed rats suppressed fasting-induced increases in food intake. However, down-regulation of KLF4 in the ARC of HFD-fed rats did not reverse HFD-induced leptin resistance. Overall, these findings demonstrate that KLF4 participates in the physiological regulation of feeding by mediating the effects of leptin.

## 2 Material and methods

### 2.1 Animals

For all experiments, 8- to 10-week-old (250–300 g) male Sprague Dawley rats were housed in individual cages under conditions of controlled temperature (23 °C) and illumination (12 h light/12 h dark cycle). They had *ad libitum* access to water and standard laboratory chow, unless otherwise stated. For all surgical procedures rats were anesthetized by an intraperitoneal (IP) injection of ketamine–xylacine anaesthetics (80 mg/kg and 8 mg/kg of body weight, respectively). To determine how food deprivation influenced KLF4 protein levels, three groups of animals were used: a control group was fed *ad libitum*, an two additional experimental groups were deprived of food for 12 h or 48 h. To study the effect of fasting-induced food intake on hypothalamic KLF4 levels, an overnight fasting was performed before measuring food intake during 2 h. To study how leptin modulates KLF4 protein levels, one group of rats received an IP injection of vehicle (NaHCO_3_ 100 mM, pH 7.9) or recombinant rat leptin (1 mg/kg; provided by Dr A. F. Parlow, National Hormone and Peptide Program, Harbor-UCLA Medical Center, Torrance CA). Additionally, 8-week-old wild type (WT) and leptin-deficient (*ob/ob*) littermate mice were purchased from Charles River Laboratories International Inc., and sacrificed one week after their arrival at our animal facilities. DIO rats were fed on HFD (65% fat content, Research Diets) for 15 weeks and their body weight and food intake measured weekly. All experiments and procedures were reviewed and approved by the Ethics Committee of the University of Santiago de Compostela, in accordance with EU guidelines for the use of experimental animals.

### 2.2 Stereotaxic microinjection of adenoviral and lentiviral expression vectors

Rats were placed in a stereotaxic frame (David Kopf Instruments, Tujunga, CA) and one of the following vectors injected with a 25-gauge needle (Hamilton): either adenoviral vectors over-expressing GFP (control) or KLF4 (1 × 10^10^ PFU/ml each; SignaGen Laboratories, MD, USA); or lentiviral vectors over-expressing shRNA against KLF4, shRNA against FOX01, or GFP (control). (shRNA KLF4 clone ID: TRCN0000095370; 5.3 × 10^6^ TU/mL; shRNA FOX01 clone ID: TRCN0000054879, Sigma–Aldrich Inc., Buchs, Switzerland). The coordinates used to reach the ARC were AP: −2.8, Lat: ±0.3, DV: −10.2. The incision was closed with sutures and rats were kept warm until fully recovered.

### 2.3 Body composition, locomotor activity and indirect calorimetry

Energy expenditure, respiratory quotient (RQ) and locomotor activity were measured as previously described [25]. Body composition was measured using NMR imaging (Whole Body Composition Analyzer; EchoMRI).

### 2.4 Leptin central pathway studies

Intracerebroventricular (ICV) cannulae were implanted in the lateral ventricle as described previously [26], and after complete recovery the rats were randomly allocated into four groups. For inhibition of the STAT3 pathway, rats were fasted overnight (12 h) and then given a single ICV infusion of either vehicle (saline) or 75 pmol STAT3 peptide inhibitor (STAT3 PI, Calbiochem). 30 min later, rats received either vehicle (10 mM NaHCO_3_, pH 7.9) or 3 μg of leptin, as described previously [27,28]. For inhibition of the phosphatidylinositide 3-kinase (PI3K) pathway, a similar protocol was followed, using either vehicle (DMSO) or 1 nmol PI3K inhibitor (LY294002; Sigma–Aldrich), as described previously [29]. 19 min later, either vehicle or 3 μg of recombinant rat leptin was administered. 30 min after leptin administration, rats were sacrificed and the ARC was dissected out using micropunches and a dissecting microscope, as previously [30]. Accuracy of ARC dissection was assessed in terms of protein levels of the ARC-specific marker AgRP. Dissections of the ventromedial hypothalamus (VMH) and the lateral hypothalamus (LHA) were also performed, and their accuracy assessed by steroidogenic factor 1 (SF1) protein levels.

### 2.5 Leptin sensitivity assays

IP leptin was injected as described previously [31], at 1 mg/kg body weight, a dose reported to reduce caloric intake and body weight in rats [32]. Vehicle (100 mM NaHCO_3_, pH 7.9) was used in control animals. To study central leptin pathways and their influence on KLF4, rats were sacrificed after 90 min; to assess peripheral effects of leptin, food intake and body weight were measured 24 h after IP administration.

### 2.6 Immunohistochemistry

Diaminobenzidine (DAB) immunohistochemistry, GFP immunofluorescence and double labelling were performed as previously described [25,30]. Paraffin sections of (4 μm) were consecutively incubated with a primary antibody against KLF4 (Cell Signalling) and AgRP (Abcam). The specificity of the antibodies was demonstrated by substitution of the second primary antibody with NRS; substitution of the second primary antibody with GFAP (Dakopatts) which was localized to different cell types (astrocytes) than KLF4 or AgRP (neurons) used as the first primary antibodies that showed no colocalization of the immunofluorescence signal (data not shown).

### 2.7 *In situ* hybridization

*In situ* hybridization assays were performed to visualize hypothalamic mRNA expression of KLF4, AgRP, NPY, POMC and CART, as previously described [33].

### 2.8 Western blotting

Total protein was extracted from liver, epididymal white adipose tissue (WAT) and ARC as previously described [34]. Briefly, total protein lysates from WAT (30 μg) and ARC (12 μg) were subjected to immunoblotting, as previously described [25], and probed with the antibodies to the following: acetyl CoA carboxylase (ACC), phospho-ACC-Ser79 (pACC) (Upstate); FOX01, phospho-FOX01 (pFOX01), KLF-4, STAT3, phospho-STAT3 (pSTAT3) and phospho-hormone-sensitive lipase (Ser-660) (Cell Signalling); AgRP, glutamatic acid decarboxylase 65 (GAD65), glutamatic acid decarboxylase 67 (GAD67) and vesicular GABA transporter (VGAT) (Abcam); NPY and β-actin (Sigma–Aldrich); fatty acid synthase (FAS) (H-300), HSL, lipoprotein lipase (LPL) (H-53) and SF1 (Santa Cruz Biotechnology). 6–8 samples per animal group were used, and protein levels were normalized to β-actin for each sample.

### 2.9 Liver triglyceride content

The extraction procedure for liver triglycerides (TG) was performed as described previously [25]. TG content of each sample was measured using a colorimetric assay (Spinreact).

### 2.10 Statistical analysis and data presentation

Data are expressed as mean ± SEM. mRNA and protein data are expressed in relation (%) to control (vehicle-treated) rats. Statistical significance was determined using Student's *t*-test to compare two groups. *p* < 0.05 was considered significant. Two-way ANOVA was used to examine interactions between variables when two factors were analysed.

## 3 Results

### 3.1 Nutritional status and leptin regulate KLF4 protein levels in the hypothalamic arcuate nucleus

**Figure 1:**
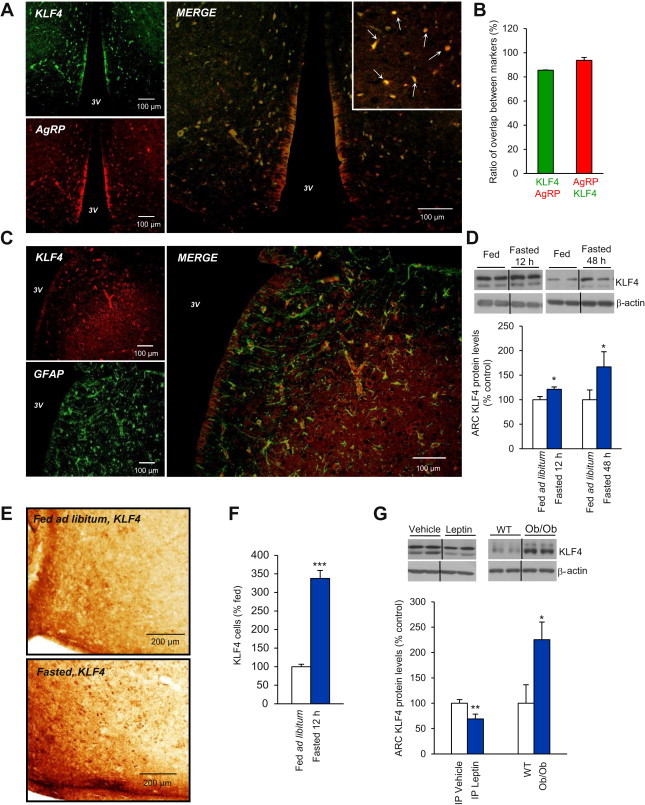
Colocalization (merge) of KLF4 (green) and AgRP (red) neurons in the hypothalamic arcuate nucleus (ARC) (A). Quantification of co-expression of KLF4 and AgRP shows that 85 ± 0.2% of KLF4 neurons expressed AgRP (green bar) and that 93 ± 2% of AgRP neurons expressed KLF4 (red bar) (B). Colocalization of KLF4 (red) and GFAP (green) in the ARC (C). Effect of 12 h and 48 h of fasting on KLF4 protein levels in the ARC measured by western blot (D) and immunohistochemistry (E). Quantification of cells expressing KLF4 in the ARC of fasted rats (337 ± 21%) compared the total KLF4 neurons in *ad libitum* rats (F). Effect of IP leptin treatment and leptin deficiency on KLF4 protein levels in the ARC measured by western blot (G). Dividing lines indicate splicings of the same gel. Values are mean ± SEM of 8–10 animals per group. β-actin was used to normalize protein levels. Error bars indicate SEM. *p < 0.05, **p < 0.01 and ***p < 0.001 versus controls using one-tailed Student's *t*-test.

KLF4 and AgRP were colocalized in the hypothalamic arcuate nucleus (ARC) (Figure 1A). Quantification of co-expression of KLF4 and AgRP shows that 85 ± 0.2% of KLF4 neurons expressed AgRP and that 93 ± 2% of AgRP neurons expressed KLF4 (Figure 1B). However, KLF4 and GFAP did not show colocalization in the ARC (Figure 1C). We first hypothesized that if KLF4 binds to a specific CACCC-box in the AgRP promotor [8], hypothalamic KLF4 should be modulated by nutritional status and leptin. Using *in situ* hybridization we observed that KLF4 mRNA expression was expressed in the hypothalamic ARC (Supplementary Figure 1A). To further assess the relevance of this finding we dissected out the ARC and assessed its protein levels. ARC and VMH dissections were corroborated by protein levels of their specific markers, AgRP in the ARC (Supplementary Figure 1B) and SF1 in the VMH (Supplementary Figure 1C). Colocalization studies of AgRP and KLF4 showed a high degree of neuronal co-expression in the ARC (Figure 1A and B), whereas KLF4 and GFAP were not colocalized within the same hypothalamic area (Figure 1C). Western blotting showed that within the ARC, KLF4 protein levels were increased after 12 h and 48 h fasting as compared with rats fed *ad libitum* (Figure 1D). These data were corroborated by immunohistochemistry (Figure 1E), with an increased intensity of immunostaining being observed in the ARC of fasted rats as compared with rats fed *ad libitum* (Figure 1F).

**Figure 2:**
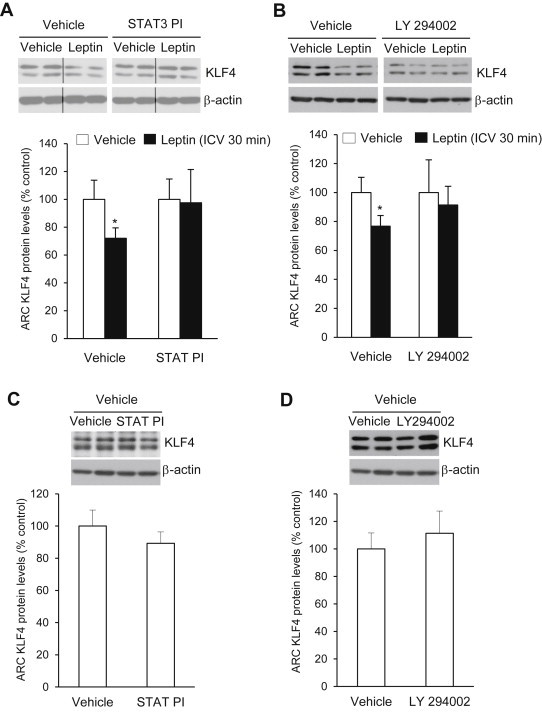
KLF4 protein levels in the hypothalamic arcuate nucleus of rats treated with a STAT3 inhibitor (A) or PI3K inhibitor (B) plus vehicle or leptin. Effect of the STAT3 inhibitor (C) or PI3K inhibitor (D) on KLF4 protein levels in the hypothalamic arcuate nucleus. Dividing lines indicate splicings of the same gel. Values are mean ± SEM of 8–10 animals per group. Error bars indicate SEM. *p < 0.05 versus controls using one-tailed student's *t*-test.

Because fasting is a hypoleptinemic state, we next tested whether leptin, one of the predominant hormones known to inhibit the activity of AgRP neurons, affected hypothalamic KLF4 levels. We found that IP administration of leptin decreased KLF4 levels in the ARC after 90 min in rats, but, by contrast, in leptin-deficient (*ob/ob*) mice ARC KLF4 protein levels were significantly increased (Figure 1G). We hypothesized that these effects were mediated by STAT3 and/or PI3K, since leptin seems to be an important modulator of hypothalamic KLF4 and both the STAT3 and PI3K signalling pathways are essential modulators of the anorectic action of leptin [35]. To address the role of central STAT3 signalling in mediating the effects of leptin on KLF4, we used a cell-permeable phosphopeptide-specific inhibitor of the STAT3 signalling pathway (STAT3 PI), which is normally activated by the long form of the leptin receptor [27]. Rats received, first, a single ICV infusion of either vehicle or STAT3 PI, and 30 min later, a second ICV infusion of either vehicle or leptin. Inhibition of the STAT3 signalling pathway in this way prevented the decrease in KLF4 protein levels caused by leptin in controls in the ARC (Figure 2A). Inhibition of the PI3K signalling pathway using the PI3K inhibitor LY294002 [28,29] similarly prevented leptin-induced suppression of KLF4 protein expression (Figure 2B). Importantly, the inhibition of STAT3 (Figure 2C) or PI3K (Figure 2D) did not themselves cause any alteration in KLF4 protein levels in the ARC.

### 3.2 Over-expression of KLF4 in the hypothalamic arcuate nucleus increases food intake and body weight through stimulation of AgRP

**Figure 3:**
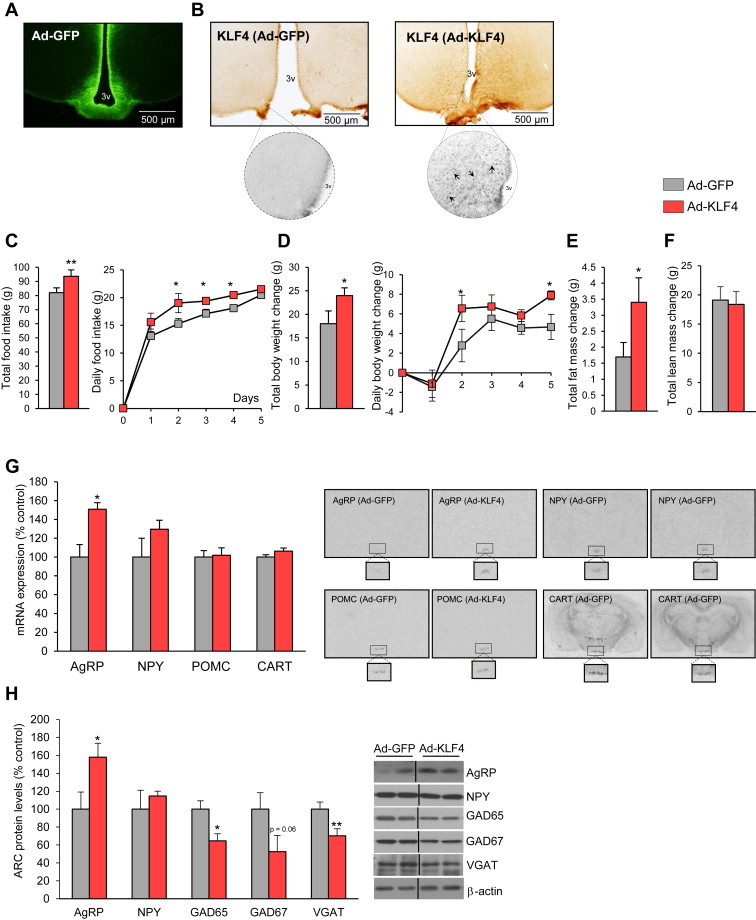
GFP in the hypothalamic arcuate nucleus (A). KLF4 protein levels in the hypothalamic arcuate nucleus in Ad-GFP and Ad-KLF4 treated rats (B). Effect of the over-expression of KLF4 in the hypothalamic arcuate nucleus on food intake (C), body weight (D), fat mass (E), lean mass (F), hypothalamic mRNA expression of NPY, AgRP, POMC and CART (G), and arcuate protein levels of AgRP, NPY, GAD65, GAD67 and VGADT (H). Dividing lines indicate splicings of the same gel. Values are mean ± SEM of 8–10 animals per group. Error bars indicate SEM. *p < 0.05 and **p < 0.01 versus controls using one-tailed student's *t*-test.

Since KLF4 is located in AgRP neurons and is regulated by nutritional status and leptin, we next investigated whether specific over-expression of KLF4 in the ARC would be sufficient to affect food intake and body weight. Stereotaxic injection of adenoviruses over-expressing KLF4 into the ARC (Figure 3A) showed that the over-expression of KLF4 in this specific nucleus (Figure 3B) increased total food intake (Figure 3C) and body weight (Figure 3D) over a 5-day period. These transient effects on food intake and body weight are probably explained by the limited length of action of the adenoviruses. The gain of weight was consistent with an increased fat mass (Figure 3E) without any significant change in lean mass (Figure 3F). These effects were sufficiently explained by the hyperphagia, because energy expenditure, locomotor activity and respiratory quotient remained unaltered in the same period following KLF4 over-expression (Supplementary Figure 2). In rats in which adenoviral injections did not reach the ARC no differences in food intake or body weight were observed, suggesting that KLF4 acts specifically in the ARC (Supplementary Figure 3). Consistent with their increase in fat mass, rats injected in the ARC with adenoviruses over-expressing KLF4 also showed larger adipocytes (Supplementary Figure 4A) and higher levels of FAS and lipoprotein (LPL) in WAT than rats injected with empty viruses (Supplementary Figure 4B). No differences in hepatic TG levels were detected between the two groups (Supplementary Figure 4C).

To ascertain which hypothalamic neuropeptides were triggered by over-expression of KLF4, we next assessed ARC levels of AgRP, NPY, POMC and CART. Thus, we found that the over-expression of KLF4 produced a significant up-regulation of AgRP mRNA expression, whereas other key neuropeptides regulating food intake, such as CART, NPY and POMC, were not affected (Figure 3G). These data, obtained by *in situ* hybridization, were corroborated by western blotting, with a significant increase being observed in AgRP but not NPY protein levels (Figure 3H). Additionally, we also found that the levels of GABA synthesizing enzymes glutamate decarboxylase 65 and 67 (GAD65 and GAD67) and vesicular GABA transporter (VGAT) were decreased in the ARC after over-expression of KLF4 (Figure 3H).

### 3.3 Activation of KLF4 in the hypothalamic arcuate nucleus blunts leptin sensitivity in rats fed a chow diet

**Figure 4:**
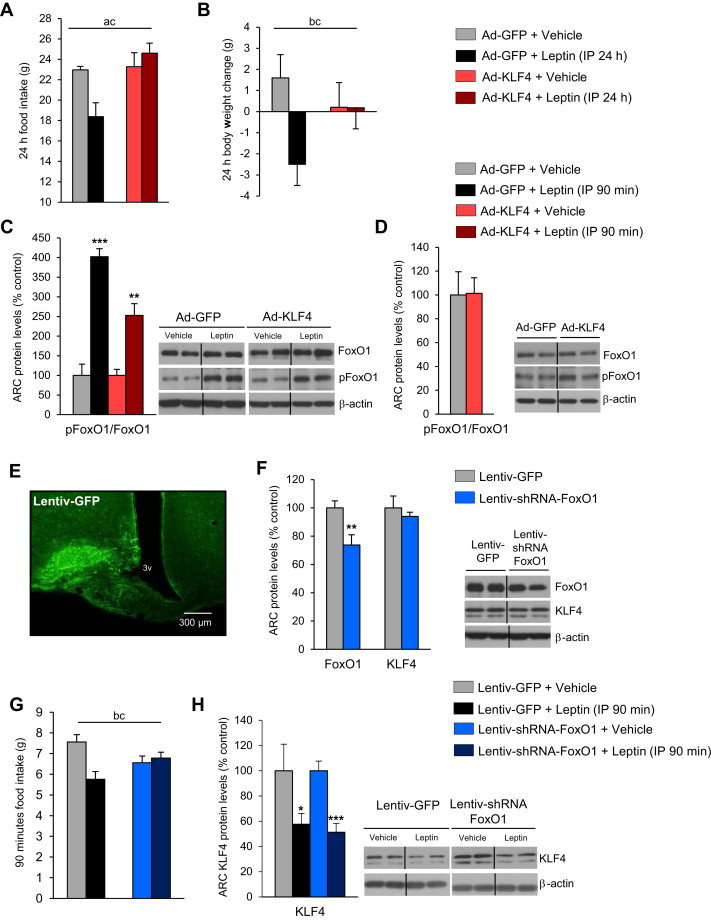
Effect of peripheral leptin (1 mg/kg) or vehicle administration on rats after 4-day adenoviral injection expressing GFP or KLF4 respectively on food intake (A) and body weight (B) after 24 h. Effect of peripheral leptin (1 mg/kg) or vehicle administration on rats after 4-day adenoviral injection expressing GFP or KLF4 on FoxO1 levels in the hypothalamic arcuate nucleus (ARC) after 90 min (C). Effect of over-expression of KLF4 in the ARC on FoxO1 protein levels in the ARC (D). Localization studies of microinjection sites, showing immunofluorescence with anti-GFP antibody in the ARC (E). Protein levels of FoxO1 and KLF4 in the ARC of rats stereotaxically injected with scrambled or shFoxO1 lentiviruses (F). Effect of peripheral leptin (1 mg/kg) or vehicle administration on rats after 21-days of the lentiviral injection expressing GFP or FoxO1 shRNA on food intake (G) and KLF4 protein levels in the ARC (H) after 90 min of leptin injection. Dividing lines indicate splicings of the same gel. β-actin was used to normalize protein levels. Error bars indicate SEM. Annotation indicates significant effect of a = virus delivery, b = leptin infusion or c = significant virus–leptin interaction, defined as p < 0.05 using two-way ANOVA. *p < 0.05; **p < 0.01 versus controls using one-tailed student's *t*-test.

Given the data thus far, showing KLF4 to be regulated by leptin and to stimulate both *in vivo* AgRP expression and food intake, we next hypothesized that hypothalamic KLF4 modulates leptin sensitivity. To test this, we used an adenovirus to over-express KLF4 in the ARC for 4 days and then delivered IP leptin or its vehicle, following a published protocol [31]. As expected, in control rats treated with the empty virus the subsequent peripheral administration of leptin decreased 24 h-food intake (Figure 4A) and body weight (Figure 4B). However, over-expression of KLF4 in the ARC completely blocked leptin's subsequent actions on both feeding and body weight (Figure 4A and B).

FoxO1 is a transcription factor that directly regulates AgRP expression [20], and its genetic ablation in AgRP neurons leads to reduced food intake and fat mass [21]. FoxO1 is, moreover, a critical modulator of leptin sensitivity [21,36]. Therefore, we next evaluated any potential relationship between FoxO1 and KLF4 in modulating leptin sensitivity. As expected, after 90 min peripheral administration of leptin increased phosphorylation of FoxO1 (pFoxO1) in the ARC of control rats (Figure 4C). Similarly, leptin also triggered pFoxO1 in rats over-expressing KLF4 in the ARC (Figure 4C), indicating that manipulation of KLF4 had not altered FoxO1 activity. Consistently, over-expression of KLF4 in the ARC did not modify FoxO1 protein levels in the ARC (Figure 4D). Next, we examined the effect of FoxO1 down-regulation in the ARC on leptin sensitivity, using lentiviral FoxO1 shRNA stereotaxically injected into the ARC and leptin administered peripherally. The efficiency of the stereotaxic injections in the ARC was corroborated by immunostaining of GFP (Figure 4E). FoxO1 protein levels were indeed decreased, whereas KLF4 protein levels remained unaffected in the ARC after the stereotaxic injection of the FoxO1 shRNA lentiviruses, (Figure 4F). The inhibition of FoxO1 blunted the anorexigenic action of leptin (Figure 4G). However, as shown in Figure 4G, leptin decreased KLF4 protein levels in the ARC of both control rats and in rats injected with lentiviral FoxO1 shRNA (Figure 4H).

### 3.4 Hypothalamic KLF4 does not mediate leptin resistance in diet-induced obesity

**Figure 5:**
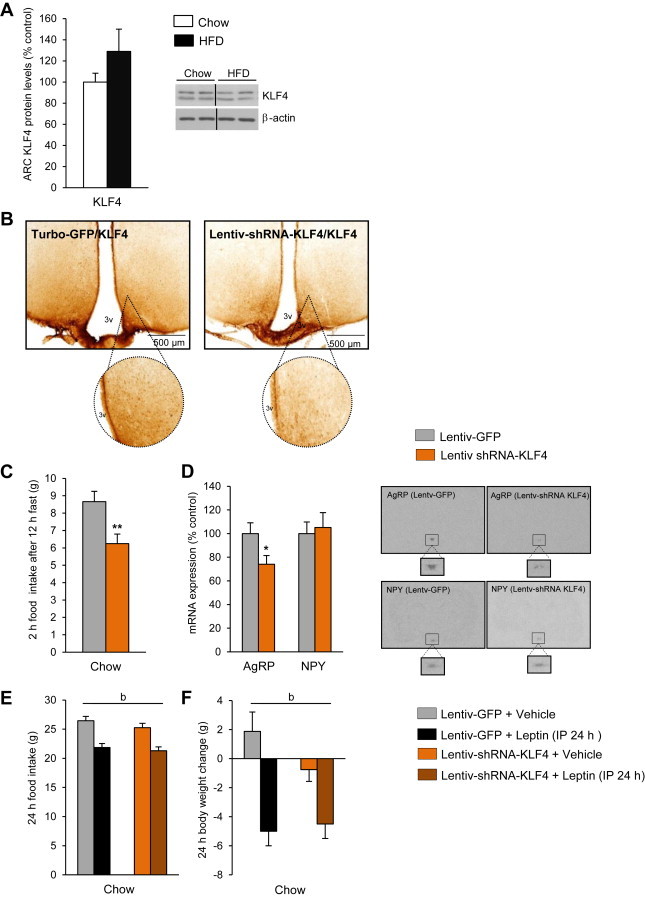
KLF4 protein levels in the hypothalamic arcuate nucleus (ARC) of rats fed a chow diet and high-fat diet (A). KLF4 protein levels in the ARC in lentivirus-GFP and lentivirus-shRNA KLF4 treated rats (B). Effect of ARC KLF4 down-regulation on 2 h food intake after 12 h of fasting (C) and hypothalamic levels of AgRP and NPY (D) in rats fasted for 12 h and fed a chow diet for 2 h. Effect of peripheral leptin (1 mg/kg) or vehicle administration on rats after 21 days of injection with a lentivirus expressing GFP or KLF4 shRNA on food intake (E) and body weight (F) of rats fed a chow diet. Dividing lines indicate splicings of the same gel. Values are mean ± SEM of 8–10 animals per group. β-actin was used to normalize protein levels. Error bars indicate SEM. Annotation indicates significant effect of a = virus delivery, b = leptin infusion or c = significant virus–leptin interaction, defined as *p* < 0.05 using two-way ANOVA. *p < 0.05; **p < 0.01 versus controls using one-tailed student's *t*-test.

A hallmark of HFD-induced obesity is leptin resistance. Leptin signalling in the hypothalamus is blunted in rats fed a HFD [37]. Since KLF4 is a key modulator of leptin's actions on food intake and body weight, we next hypothesized that hypothalamic KLF4 contributes to the development of HFD-induced leptin resistance. To test this, we initially measured KLF4 protein levels in the ARC of rats fed a chow diet and rats fed a HFD (Figure 5A) but we failed to detect significant differences between these two groups. Then, we used a lentivirus encoding a KLF4 shRNA to inhibit expression of KLF4 specifically within the ARC (Figure 5B). We predicted that chronic inhibition of KLF4 would restore leptin sensitivity in HFD-fed leptin-resistant rats. First, we demonstrated that the lentiviral KLF4 shRNA was able to inhibit the hyperphagic response in rats pre-fasted overnight when the animals were fed a chow diet (Figure 5C). The reduced hyperphagia was consistent with a specific decrease in AgRP mRNA expression (Figure 5D). Chronic inhibition of KLF4 in the ARC of rats fed a chow diet did not cause any alteration in cumulative food intake, body weight or fat mass (Supplementary Figure 5A and B). However, weekly measurements indicated that there was a significant decrease in the body weight gain after the third week (Supplementary Figure 5A and B). In order to investigate if the down-regulation of KLF4 in the ARC was able to modulate leptin sensitivity, we injected intraperitoneally leptin in rats 3 weeks after the stereotaxic delivery of lentiviral KLF4 shRNA into the ARC of rats fed a chow diet. However, our data indicated that the lower levels of KLF4 in the ARC did not modify leptin sensitivity, since rats injected with empty lentiviruses showed similar food intake (Figure 5E) and body weight (Figure 5F) to rats injected with lentiviral KLF4 shRNA.

**Figure 6:**
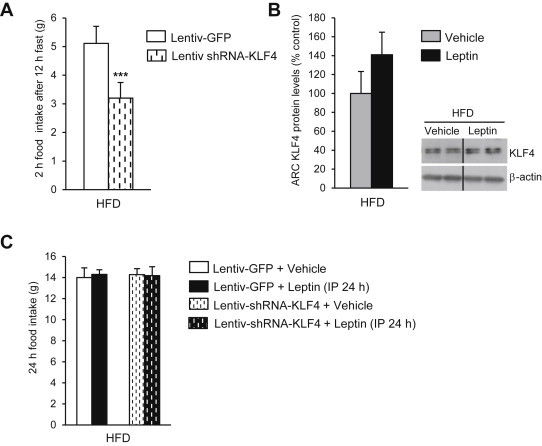
Effect of KLF4 down-regulation on 2 h food intake after 12 h of fasting (A). KLF4 protein levels in the ARC of DIO rats treated with leptin (B). Effect of peripheral leptin (1 mg/kg) or vehicle administration on rats treated with lentivirus-GFP and lentivirus shRNA KLF4 in rats fed a high fat diet (HFD) (C). Dividing lines indicate splicings of the same gel. Values are mean ± SEM of 8–10 animals per group. β-actin was used to normalize protein levels. Error bars indicate SEM. *p < 0.05; **p < 0.01; ***p < 0.001 versus controls using one-tailed student's *t*-test.

Finally, to study the role of KLF4 in DIO rats, we injected lentiviral KLF4 shRNA into the ARC of HFD-fed rats. Similar to a chow diet, rats fed a HFD and then administered lentiviral KLF4 shRNA (three weeks after one single lentiviral injection) showed a decreased hyperphagic response after overnight pre-fasting (Figure 6A). Chronic inhibition of KLF4 in the ARC of HFD-fed rats also did not cause any alteration in food intake, body weight or fat mass (Supplementary Figure 5C and D). When we challenged DIO rats with exogenous IP leptin at a dose that reduced caloric intake and body weight in lean rats, leptin failed to inhibit hypothalamic KLF4 expression (Figure 6B). To explore whether silencing KLF4 in the ARC altered whole-body leptin sensitivity, we measured food intake 24 h after IP leptin administration. As expected, leptin failed to suppress food intake in DIO rats treated with the empty lentivirus. We also found that DIO rats treated with the lentiviral KLF4 shRNA remained leptin resistant (Figure 6C).

## 4 Discussion

This study establishes the relevance of KLF4 as an *in vivo* transcription factor involved in energy homeostasis. More specifically, two lines of evidence support this conclusion: first, hypothalamic KLF4 is regulated by nutritional status and by leptin in a FoxO1-independent manner; and second, viral-mediated over-expression of KLF4 triggers AgRP levels and blunts the anorectic action of leptin in lean rats.

The importance of AgRP neurons in the control of energy balance has led to several investigations of their mechanisms of action, projections to and from other neural populations, and the signalling pathways that modulate AgRP expression [38]. The transcription factor KLF4 is one of the most recently discovered activators of AgRP. In vitro studies involving over-expression or silencing of KLF4 have shown that KLF4 is required for activation of AgRP [8,39]. *In vivo*, a compound named PMI-5011 has also been found to activate both KLF4 and AgRP expression; however, PMI-5011 also activated hypothalamic orexin and melanin-concentrating hormone, stimulating food intake by that route [8]. Two important issues have remained unanswered: first, in which cell types KLF4 is located within the hypothalamus and, second, whether specific manipulation of KLF4 *in vivo* is sufficient to control AgRP activity and AgRP-stimulated food intake, nutrient partitioning and body weight [26,40,41]. We now report that KLF4 is predominantly located in the ARC and, within this hypothalamic site, is localized to AgRP neurons. Consistent with its previously reported regulation of AgRP expression [42], our data also show that KLF4 protein levels in the ARC are increased after fasting and down-regulated by leptin.

To further test the capacity of KLF4 to modulate AgRP *in vivo*, we injected viral vectors that over-express KLF4 into the ARC, and found that animals carrying these viruses ate more and gained more weight than their controls, but that neither energy expenditure nor respiratory quotient changed significantly. This KLF4-induced food intake could only be explained by increased levels of AgRP, because levels of the other known relevant neuropeptides in the ARC, namely NPY, CART or POMC, were unaffected by KLF4 over-expression. Interestingly, we found that the levels of the GABA synthesizing enzymes GAD65 and GAD67 as well as the vesicular GABA transporter (VGAT) were decreased in the ARC after over-expression of KLF4. Since mice lacking vesicular GABA transporter in AgRP neurons are lean and resistant to obesity [43], we initially expected that the over-expression of KLF4 would increase the synthesis and/or transport of GABA. However, the decreased levels of GAD65, GAD67 and VGAT observed in the ARC after over-expression of KLF4 suggest that: a) KLF4 actions on feeding and body weight do not involve GABA signalling; and b) the decrease in synthesis and transport of GABA following over-expression of KLF4 might be due to a compensatory response. In addition, administering viral vectors that silenced KLF4 in the ARC did not elicit differences in either food intake or body weight, and the fasting-induced response seen in control animals was blunted. Moreover, this impaired response to fasting was specifically mediated by AgRP, since the silencing of KLF4 caused a significant decrease in fasting-induced hypothalamic AgRP levels. Importantly, fasting-induced food intake was compromised in both lean and DIO rats, indicating that KLF4 is an important modulator of the normal fasting response, independent of the type of diet. Taken together, these new data support previous *in vitro* results [8,9] and indicate that KLF4 is a specific activator of AgRP neurons that modulates its biological actions *in vivo*.

AgRP neurons were previously known to play a critical role in mediating the actions of leptin [44]. It was also thought that increased levels of hypothalamic KLF4 induced by fasting, a hypoleptinemic state, were probably regulated by leptin levels. Specifically, the inhibition of KLF4 caused by leptin was known to be mediated by both STAT3 and PI3K, two key modulators of its anorectic action [35]. Those data thus indicated that KLF4 was part of the leptin signalling pathway, and that it was situated downstream of STAT3 and PI3K. In order to investigate the functional role of KLF4 in leptin's action, we therefore challenged leptin in rats injected either with an empty virus or with a viral vector over-expressing KLF4, into the ARC. Our results indicate that activation of KLF4 in the ARC is sufficient to abrogate the anorectic effect of leptin in lean rats, and therefore that KLF4 situated in the ARC is an essential component of the leptin signalling pathway that controls food intake. Since FoxO1 mediates AgRP-dependent effects of leptin on food intake [20] we hypothesized that KLF4 might be interacting with FoxO1. However, leptin was still able to increase FoxO1 in the ARC of rats injected with the viral vector over-expressing KLF4, and also able to stimulate KLF4 when FoxO1 was inhibited in the ARC. Thus, our results collectively indicate that KLF4 is not a direct target of FoxO1, and suggest that each transcription factor functions independently to modulate leptin sensitivity.

CNS resistance to leptin is likely to be an early contributor to the weight gain associated with DIO [45], and decreased leptin signalling has been demonstrated in DIO rodents [37,46]. We therefore hypothesized that decreasing KLF4 in the ARC of DIO rats would be able to reverse HFD-induced leptin resistance. Consistent with that hypothesis, we found that peripherally administered leptin failed to decrease hypothalamic KLF4 levels in DIO rats, and this could explain the lack of effect that leptin had on feeding behaviour in DIO rats. Unexpectedly, however, leptin was not able to reduce food intake in rats treated with a viral vector silencing KLF4 in the ARC. These latter data show that KLF4 does not ameliorate HFD-induced peripheral leptin resistance. The potential explanations for this effect might be that leptin resistance is mediated by a variety of factors including: a) alterations in leptin transport across the blood–brain barrier; b) alterations in leptin receptor gene expression, and endocytosis and trafficking of ligand-activated cell surface receptors; and c) alterations in the leptin signalling pathway [47–49]. Therefore, we cannot entirely rule out the possibility that KLF4 may be able to restore some of the components, albeit not all, involved in HFD-induced leptin resistance and that still represents a potential target to restore neuronal leptin sensitivity.

## 5 Conclusions

a) Hypothalamic KLF4 is regulated by leptin through both STAT3 and PI3K signalling pathways.
b) KLF4 over-expression in the ARC is sufficient to increase food intake and to blunt the anorectic action of leptin in a FoxO1-independent manner.
c) Leptin fails to inhibit hypothalamic KLF4 expression in DIO rats, while KLF4 does not on its own regulate HFD-induced peripheral leptin resistance.

## Appendix A Supplementary data

**Supplemental Figure 1.** Representative in situ hybridization of KLF4 in rat brain (A). Corroboration of specific hypothalamic nuclei isolation. AgRP protein levels in the ARC or LHA (B). SF1 protein levels in the ARC or VMH (C).

**Supplemental Figure 2.** Effect of a 4-day adenoviral injection expressing GFP or KLF4 respectively on ARC on energy expenditure (A), locomotor activity (B) and respiratory quotient (C). Values are mean ± SEM of 8–10 animals per group.

**Supplemental Figure 3.** Lack of effect of a 7-day adenoviral injection expressing GFP or KLF4 respectively in a non-specific area (A) on food intake (B) and body weight (C). Values are mean ± SEM of 8–10 animals per group.

**Supplemental Figure 4.** Effect of a 4-day adenoviral injection expressing GFP or KLF4 respectively in ARC on WAT adipocyte area (A), WAT ACC, pACC, FAS, LPL, HSL and pHSL protein levels (B), and hepatic triglicerides (TG) content represented by red oil and total TG content (C). Values are mean ± SEM of 8–10 animals per group. **p* < 0.05; ***p* < 0.01 versus controls.

**Supplemental Figure 5.** Effect of KLF4 down-regulation (using lentiviral shKLF4 injection) on food intake (A) and body weight (B) of rats fed a chow diet and food intake (C) and body weight (D) of DIO rats. Values are mean ± SEM of 8–10 animals per group. **p* < 0.05 versus controls.

## References

[1] Shields J.M., Christy R.J., Yang V.W. (1996). Identification and characterization of a gene encoding a gut-enriched Kruppel-like factor expressed during growth arrest. Journal of Biological Chemistry.

[2] Shields J.M., Yang V.W. (1998). Identification of the DNA sequence that interacts with the gut-enriched Kruppel-like factor. Nucleic Acids Research.

[3] Mahatan C.S., Kaestner K.H., Geiman D.E., Yang V.W. (1999). Characterization of the structure and regulation of the murine gene encoding gut-enriched Kruppel-like factor (Kruppel-like factor 4). Nucleic Acids Research.

[4] Segre J.A., Bauer C., Fuchs E. (1999). Klf4 is a transcription factor required for establishing the barrier function of the skin. Nature Genetics.

[5] Black A.R., Black J.D., Azizkhan-Clifford J. (2001). Sp1 and Kruppel-like factor family of transcription factors in cell growth regulation and cancer. Journal of Cellular Physiology.

[6] Qin S., Liu M., Niu W., Zhang C.L. (2011). Dysregulation of Kruppel-like factor 4 during brain development leads to hydrocephalus in mice. Proceedings of the National Academy of Sciences of the United States of America.

[7] Moore D.L., Blackmore M.G., Hu Y., Kaestner K.H., Bixby J.L., Lemmon V.P. (2009). KLF family members regulate intrinsic axon regeneration ability. Science.

[8] Ilnytska O., Stutz A.M., Park-York M., York D.A., Ribnicky D.M., Zuberi A. (2011). Molecular mechanisms for activation of the agouti-related protein and stimulation of appetite. Diabetes.

[9] Park M., Oh H., York D.A. (2009). Enterostatin affects cyclic AMP and ERK signaling pathways to regulate agouti-related protein (AgRP) expression. Peptides.

[10] Morton G.J., Cummings D.E., Baskin D.G., Barsh G.S., Schwartz M.W. (2006). Central nervous system control of food intake and body weight. Nature.

[11] Rossi M., Kim M.S., Morgan D.G., Small C.J., Edwards C.M., Sunter D. (1998). A C-terminal fragment of agouti-related protein increases feeding and antagonizes the effect of alpha-melanocyte stimulating hormone in vivo. Endocrinology.

[12] Gropp E., Shanabrough M., Borok E., Xu A.W., Janoschek R., Buch T. (2005). Agouti-related peptide-expressing neurons are mandatory for feeding. Nature Neuroscience.

[13] Luquet S., Perez F.A., Hnasko T.S., Palmiter R.D. (2005). NPY/AgRP neurons are essential for feeding in adult mice but can be ablated in neonates. Science.

[14] Bewick G.A., Gardiner J.V., Dhillo W.S., Kent A.S., White N.E., Webster Z. (2005). Post-embryonic ablation of AgRP neurons in mice leads to a lean, hypophagic phenotype. FASEB Journal.

[15] Aponte Y., Atasoy D., Sternson S.M. (2011). AgRP neurons are sufficient to orchestrate feeding behavior rapidly and without training. Nature Neuroscience.

[16] Krashes M.J., Koda S., Ye C., Rogan S.C., Adams A.C., Cusher D.S. (2011). Rapid, reversible activation of AgRP neurons drives feeding behavior in mice. Journal of Clinical Investigation.

[17] Qian S., Chen H., Weingarth D., Trumbauer M.E., Novi D.E., Guan X. (2002). Neither agouti-related protein nor neuropeptide Y is critically required for the regulation of energy homeostasis in mice. Molecular and Cellular Biology.

[18] Wu Q., Boyle M.P., Palmiter R.D. (2009). Loss of GABAergic signaling by AgRP neurons to the parabrachial nucleus leads to starvation. Cell.

[19] Varela L., Horvath T.L. (2012). AgRP neurons: a switch between peripheral carbohydrate and lipid utilization. EMBO Journal.

[20] Kitamura T., Feng Y., Kitamura Y.I., Chua S.C., Xu A.W., Barsh G.S. (2006). Forkhead protein FoxO1 mediates Agrp-dependent effects of leptin on food intake. Nature Medicine.

[21] Ren H., Orozco I.J., Su Y., Suyama S., Gutierrez-Juarez R., Horvath T.L. (2012). FoxO1 target Gpr17 activates AgRP neurons to regulate food intake. Cell.

[22] Sakkou M., Wiedmer P., Anlag K., Hamm A., Seuntjens E., Ettwiller L. (2007). A role for brain-specific homeobox factor Bsx in the control of hyperphagia and locomotory behavior. Cell Metabolism.

[23] Mesaros A., Koralov S.B., Rother E., Wunderlich F.T., Ernst M.B., Barsh G.S. (2008). Activation of Stat3 signaling in AgRP neurons promotes locomotor activity. Cell Metabolism.

[24] Fujimoto W., Shiuchi T., Miki T., Minokoshi Y., Takahashi Y., Takeuchi A. (2007). Dmbx1 is essential in agouti-related protein action. Proceedings of the National Academy of Sciences of the United States of America.

[25] Imbernon M., Beiroa D., Vazquez M.J., Morgan D.A., Veyrat-Durebex C., Porteiro B. (2013). Central melanin-concentrating hormone influences liver and adipose metabolism via specific hypothalamic nuclei and efferent autonomic/JNK1 pathways. Gastroenterology.

[26] Nogueiras R. (2007). The central melanocortin system directly controls peripheral lipid metabolism. Journal of Clinical Investigation.

[27] Buettner C., Pocai A., Muse E.D., Etgen A.M., Myers M.G., Rossetti L. (2006). Critical role of STAT3 in leptin's metabolic actions. Cell Metabolism.

[28] Buettner C., Muse E.D., Cheng A., Chen L., Scherer T., Pocai A. (2008). Leptin controls adipose tissue lipogenesis via central, STAT3-independent mechanisms. Nature Medicine.

[29] Morrison C.D., Morton G.J., Niswender K.D., Gelling R.W., Schwartz M.W. (2005). Leptin inhibits hypothalamic Npy and Agrp gene expression via a mechanism that requires phosphatidylinositol 3-OH-kinase signaling. American Journal of Physiology – Endocrinology and Metabolism.

[30] Lopez M., Varela L., Vazquez M.J., Rodriguez-Cuenca S., Gonzalez C.R., Velagapudi V.R. (2010). Hypothalamic AMPK and fatty acid metabolism mediate thyroid regulation of energy balance. Nature Medicine.

[31] Ryan K.K., Li B., Grayson B.E., Matter E.K., Woods S.C., Seeley R.J. (2011). A role for central nervous system PPAR-[gamma] in the regulation of energy balance. Nature Medicine.

[32] Wetzler S., Dumaz V., Goubern M., Tomé D., Larue-Achagiotis C. (2004). Intraperitoneal leptin modifies macronutrient choice in self-selecting rats. Physiology & Behavior.

[33] Seoane L.M., López M., Tovar S., Casanueva F.F., Señarís R., Diéguez C. (2003). Agouti-related peptide, neuropeptide Y, and somatostatin-producing neurons are targets for ghrelin actions in the rat hypothalamus. Endocrinology.

[34] Velasquez D.A., Martinez G., Romero A., Vazquez M.J., Boit K.D., Dopeso-Reyes I.G. (2011). The central sirtuin 1/p53 pathway is essential for the orexigenic action of ghrelin. Diabetes.

[35] Sahu A. (2011). Intracellular leptin-signaling pathways in hypothalamic neurons: the emerging role of phosphatidylinositol-3 kinase-phosphodiesterase-3B-cAMP pathway. Neuroendocrinology.

[36] Kim K.W., Donato J., Berglund E.D., Choi Y.H., Kohno D., Elias C.F. (2012). FOXO1 in the ventromedial hypothalamus regulates energy balance. Journal of Clinical Investigation.

[37] Frederich R.C., Hamann A., Anderson S., Lollmann B., Lowell B.B., Flier J.S. (1995). Leptin levels reflect body lipid content in mice: evidence for diet-induced resistance to leptin action. Nature Medicine.

[38] Cansell C., Denis R.G., Joly-Amado A., Castel J., Luquet S. (2012). Arcuate AgRP neurons and the regulation of energy balance. Frontiers in Endocrinology (Lausanne).

[39] Park M., Farrell J., Lemmon K., York D.A. (2009). Enterostatin alters protein trafficking to inhibit insulin secretion in beta-TC6 cells. Peptides.

[40] Joly-Amado A., Denis R.G., Castel J., Lacombe A., Cansell C., Rouch C. (2012). Hypothalamic AgRP-neurons control peripheral substrate utilization and nutrient partitioning. EMBO Journal.

[41] Cone R.D. (2005). Anatomy and regulation of the central melanocortin system. Nature Neuroscience.

[42] Mizuno T.M., Mobbs C.V. (1999). Hypothalamic agouti-related protein messenger ribonucleic acid is inhibited by leptin and stimulated by fasting. Endocrinology.

[43] Tong Q., Ye C.P., Jones J.E., Elmquist J.K., Lowell B.B. (2008). Synaptic release of GABA by AgRP neurons is required for normal regulation of energy balance. Nature Neuroscience.

[44] Varela L., Horvath T.L. (2012). Leptin and insulin pathways in POMC and AgRP neurons that modulate energy balance and glucose homeostasis. EMBO Reports.

[45] Schwartz M.W., Woods S.C., Seeley R.J., Barsh G.S., Baskin D.G., Leibel R.L. (2003). Is the energy homeostasis system inherently biased toward weight gain?. Diabetes.

[46] Howard J.K., Flier J.S. (2006). Attenuation of leptin and insulin signaling by SOCS proteins. Trends in Endocrinology & Metabolism.

[47] Caro J.F., Kolaczynski J.W., Nyce M.R., Ohannesian J.P., Opentanova I., Goldman W.H. (1996). Decreased cerebrospinal-fluid/serum leptin ratio in obesity: a possible mechanism for leptin resistance. Lancet.

[48] Mitchell S.E., Nogueiras R., Morris A., Tovar S., Grant C., Cruickshank M. (2009). Leptin receptor gene expression and number in the brain are regulated by leptin level and nutritional status. Journal of Physiology.

[49] Lopez M., Tovar S., Vazquez M.J., Nogueiras R., Seoane L.M., Garcia M. (2007). Perinatal overfeeding in rats results in increased levels of plasma leptin but unchanged cerebrospinal leptin in adulthood. International Journal of Obesity (London).

