# Supplementary material for: Hypothalamic KLF4 mediates leptin's effects on food intake via AgRP
Source: Mol Metab. 2014 Apr 15;3(4):441–51. doi: 10.1016/j.molmet.2014.04.001 (PMC4060210; doi:10.1016/j.molmet.2014.04.001)
Supplement: Supplementary file 1 [file mmc1.ppt]

## Slide 1
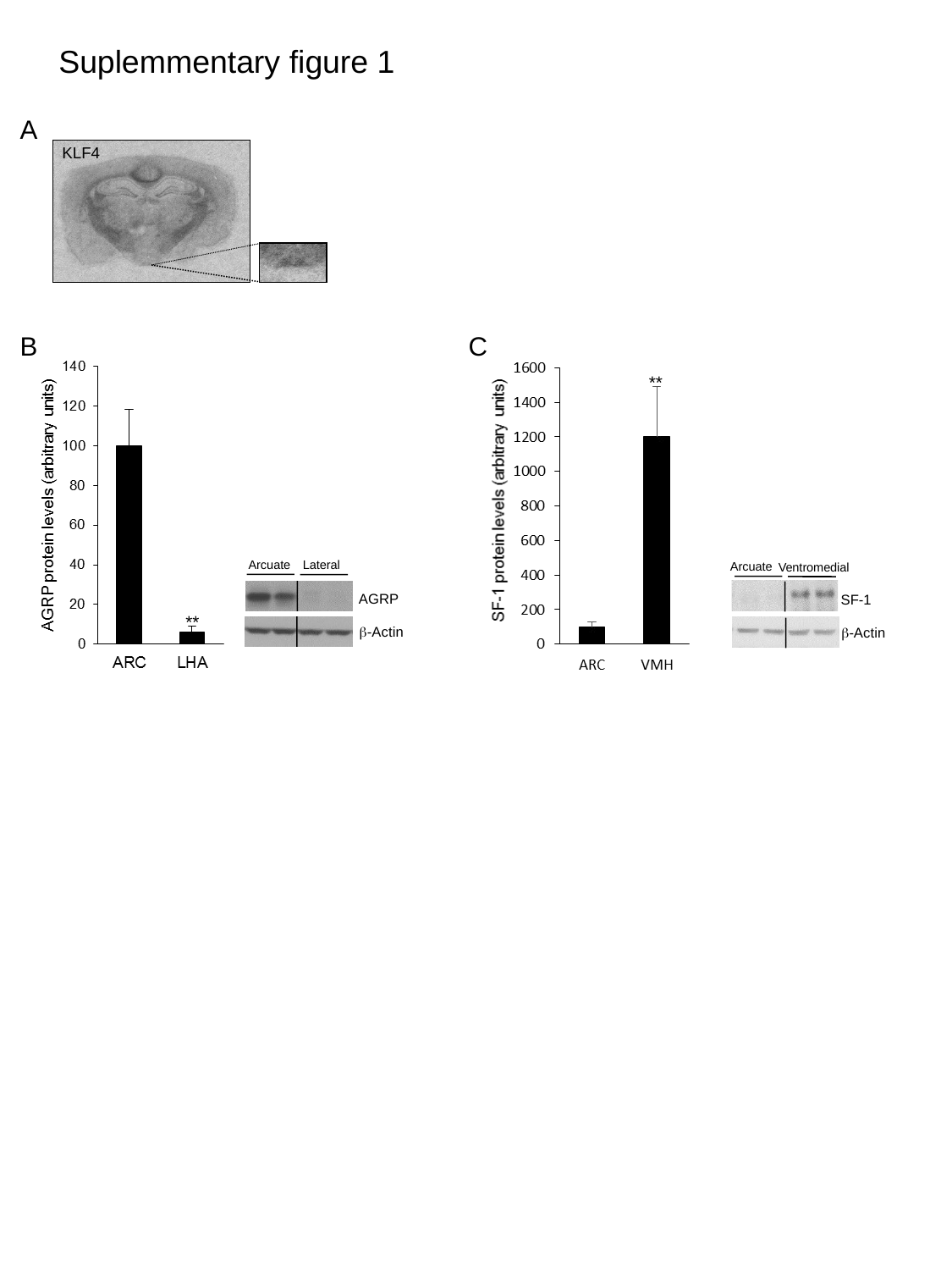

Suplemmentary figure 1
A
KLF4
B
C
**
Arcuate
Lateral
Arcuate
Ventromedial
SF-1
β-Actin
AGRP
**
β-Actin

## Slide 2
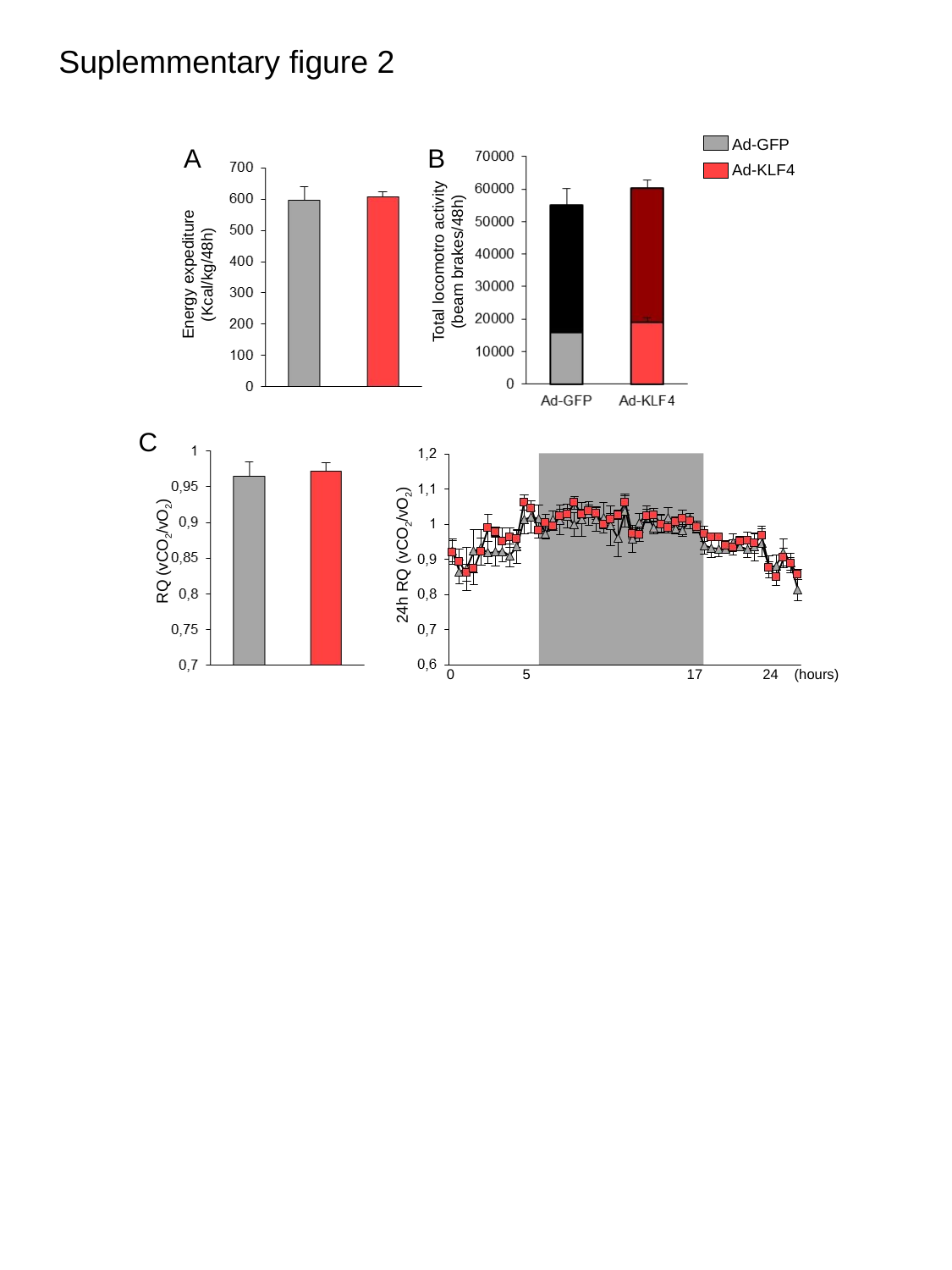

Suplemmentary figure 2
Ad-GFP
Ad-KLF4
A
B
Total locomotro activity (beam brakes/48h)
Energy expediture (Kcal/kg/48h)
C
RQ (vCO2/vO2)
24h RQ (vCO2/vO2)
0 5 17 24 (hours)

## Slide 3
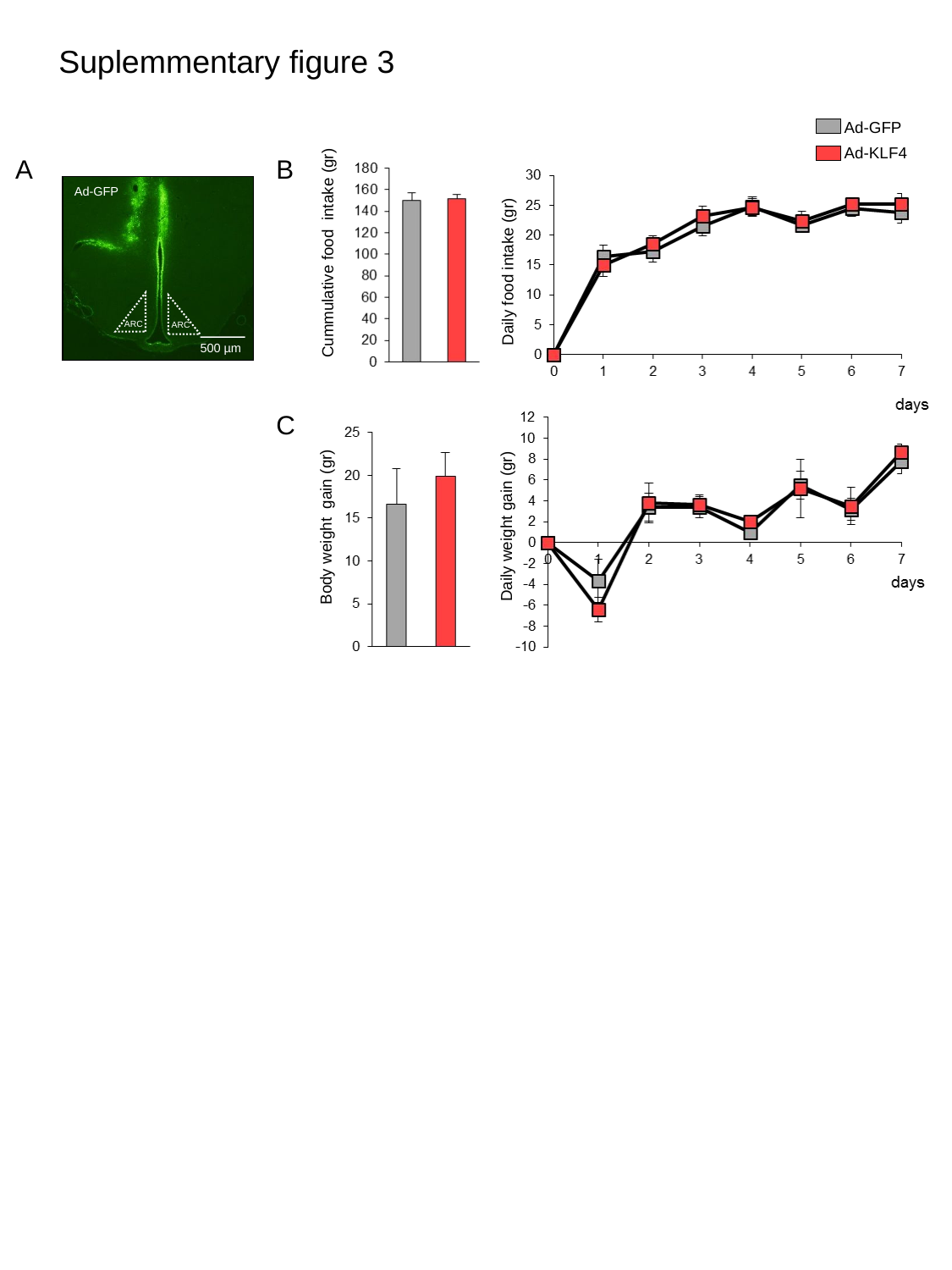

Suplemmentary figure 3
Ad-GFP
Ad-KLF4
Cummulative food intake (gr)
A
B
Daily food intake (gr)
Ad-GFP
500 µm
ARC
ARC
Daily weight gain (gr)
C
Body weight gain (gr)

## Slide 4
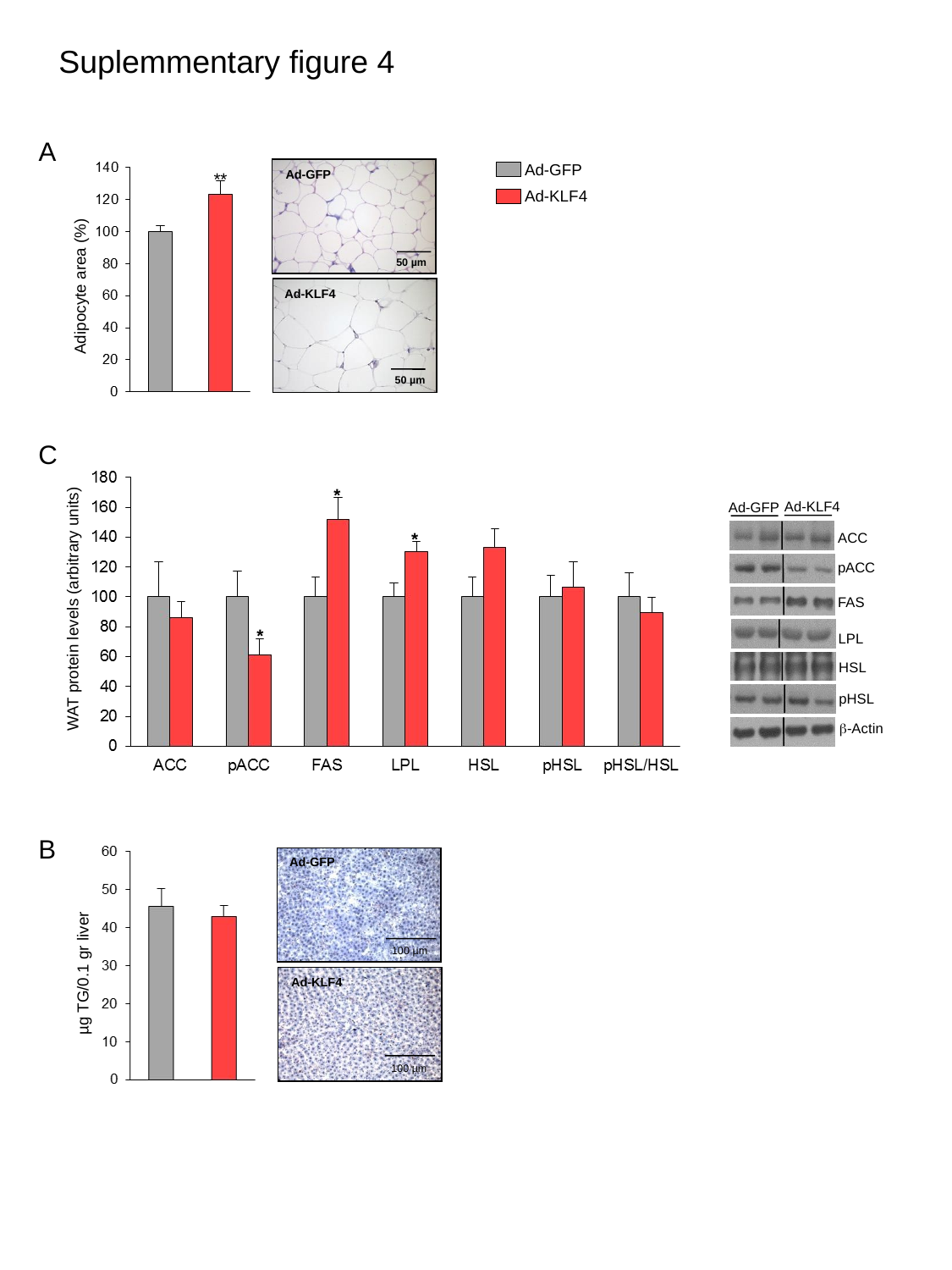

Suplemmentary figure 4
A
**
Adipocyte area (%)
Ad-GFP
Ad-KLF4
Ad-GFP
50 µm
Ad-KLF4
50 µm
C
*
*
*
WAT protein levels (arbitrary units)
Ad-KLF4
Ad-GFP
ACC
pACC
FAS
LPL
HSL
pHSL
β-Actin
B
µg TG/0.1 gr liver
Ad-GFP
100 µm
Ad-KLF4
100 µm

## Slide 5
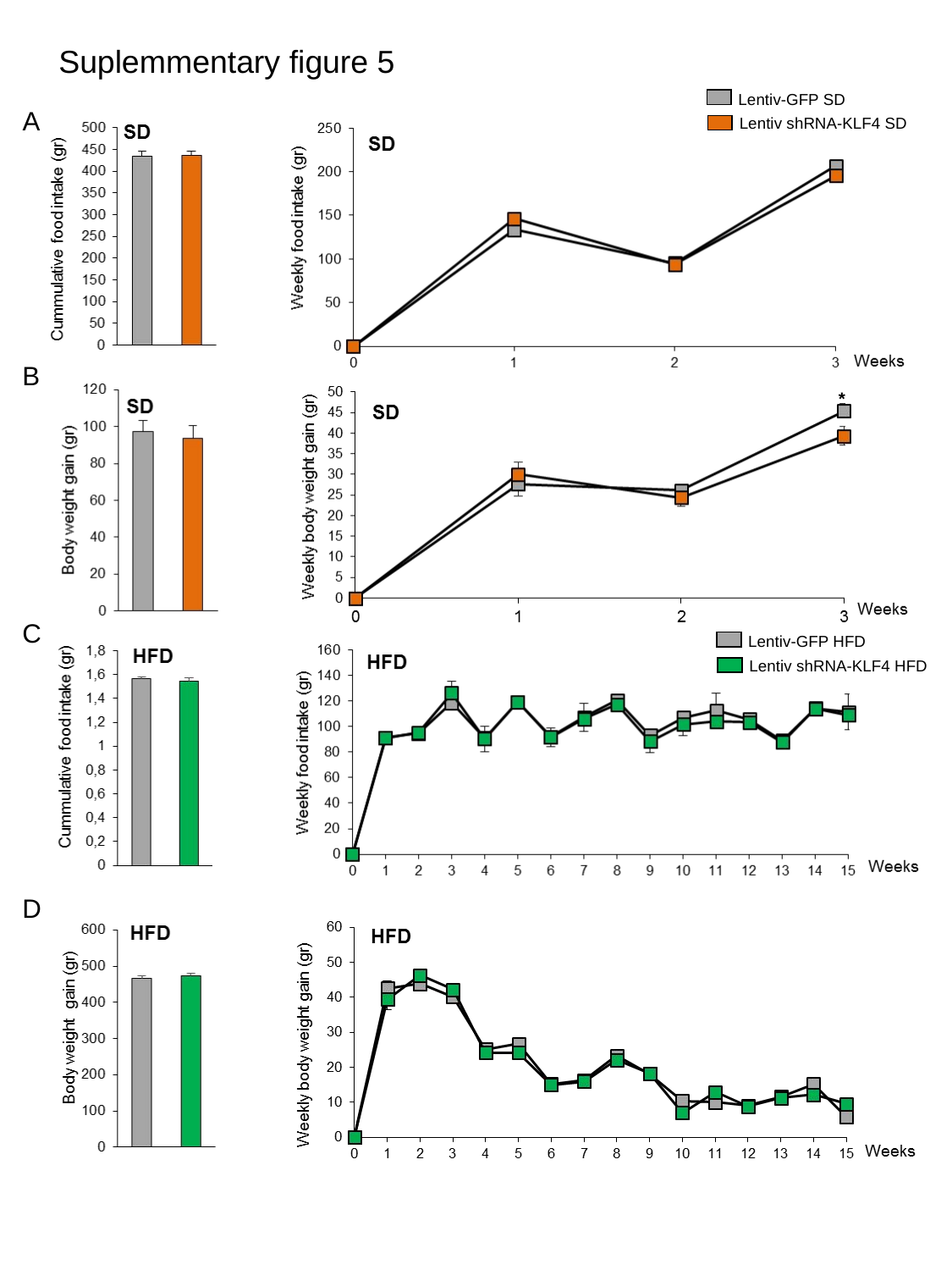

Suplemmentary figure 5
Lentiv-GFP SD
Lentiv shRNA-KLF4 SD
A
B
*
C
Lentiv-GFP HFD
Lentiv shRNA-KLF4 HFD
D
